# Darifenacin hydro­bromide

**DOI:** 10.1107/S1600536809017085

**Published:** 2009-05-14

**Authors:** S. Selvanayagam, B. Sridhar, K. Ravikumar

**Affiliations:** a Department of Physics, Kalasalingam University, Krishnankoil 626 190, India; b Laboratory of X-ray Crystallography, Indian Institute of Chemical Technology, Hyderabad 500 007, India

## Abstract

In the title compound {systematic name: (*S*)-3-[(aminocar­bonyl)diphenylmethyl]-1-[2-(2,3-di­hy­dro­benzofuran-5-yl)ethyl]pyrrolidinium bromide}, C_28_H_31_N_2_O_2_^+^·Br^−^, the pyrrolidine rings adopts an envelope conformation. The two phenyl rings make a dihedral angle of 72.5 (1)°. The four coplanar atoms of the pyrrolidine ring makes dihedral angles of 33.1 (2) and 82.8 (2)° with the two phenyl rings. The mol­ecular conformation is influenced by a C—H⋯O inter­action. In the crystal packing, there are two N—H⋯Br hydrogen bonds running in opposite directions. They appear to form *C*(10) and *C*(9) chain motifs in the unit cell. In addition, the mol­ecular packing is further stabilized by C—H⋯Br and C—H⋯O hydrogen bonds. The C atom bonded to the benzofuran ring system is disordered in a 0.66:0.34 ratio.

## Related literature

For general background to darifenacin derivatives, see: Chapple (2004 ▶); Croom & Keating (2004 ▶); Haab *et al.* (2004 ▶); Levin *et al.* (2008 ▶). For bond-length data, see: Allen *et al.* (1987 ▶); Selvanayagam *et al.* (2005 ▶). For puckering parameters, see: Cremer & Pople (1975 ▶).
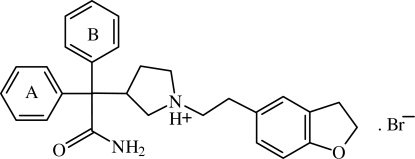


## Experimental

### 

#### Crystal data

C_28_H_31_N_2_O_2_^+^·Br^−^
*M* *_r_* = 507.46
Orthorhombic, 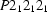

*a* = 10.2632 (7) Å
*b* = 10.9525 (8) Å
*c* = 21.7459 (16) Å
*V* = 2444.4 (3) Å^3^
*Z* = 4
Mo *K*α radiation
μ = 1.71 mm^−1^
*T* = 293 K
0.24 × 0.22 × 0.20 mm

#### Data collection

Bruker SMART APEX CCD area-detector diffractometer
Absorption correction: none
27916 measured reflections
5777 independent reflections
4703 reflections with *I* > 2σ(*I*)
*R*_int_ = 0.065

#### Refinement

*R*[*F* ^2^ > 2σ(*F* ^2^)] = 0.054
*wR*(*F* ^2^) = 0.121
*S* = 1.07
5777 reflections
315 parameters
2 restraints
H atoms treated by a mixture of independent and constrained refinement
Δρ_max_ = 0.55 e Å^−3^
Δρ_min_ = −0.84 e Å^−3^
Absolute structure: Flack (1983 ▶), 2471 Friedel pairs
Flack parameter: 0.005 (13)

### 

Data collection: *SMART* (Bruker, 2001 ▶); cell refinement: *SAINT* (Bruker, 2001 ▶); data reduction: *SAINT*; program(s) used to solve structure: *SHELXS97* (Sheldrick, 2008 ▶); program(s) used to refine structure: *SHELXL97* (Sheldrick, 2008 ▶); molecular graphics: *ORTEP-3* (Farrugia, 1997 ▶) and *PLATON* (Spek, 2009 ▶); software used to prepare material for publication: *SHELXL97* and *PARST* (Nardelli, 1995 ▶).

## Supplementary Material

Crystal structure: contains datablocks I, global. DOI: 10.1107/S1600536809017085/bt2946sup1.cif

Structure factors: contains datablocks I. DOI: 10.1107/S1600536809017085/bt2946Isup2.hkl

Additional supplementary materials:  crystallographic information; 3D view; checkCIF report

## Figures and Tables

**Table 1:** Hydrogen-bond geometry (Å, °)

*D*—H⋯*A*	*D*—H	H⋯*A*	*D*⋯*A*	*D*—H⋯*A*
N1—H1⋯Br1^i^	0.91	2.57	3.453 (6)	164
N2—H2*NB*⋯Br1^ii^	0.86 (1)	2.66 (1)	3.514 (4)	175 (5)
C18—H18⋯O2^iii^	0.93	2.60	3.382 (6)	142
C19—H19*B*⋯Br1^iv^	0.97	2.69	3.609 (5)	158
C4—H4*A*⋯Br1^iv^	0.97	2.92	3.770 (5)	147
C1—H1*B*⋯O1	0.97	2.37	2.959 (5)	119

Symmetry codes: (i) 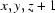; (ii) 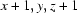; (iii) 
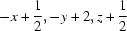
; (iv) 
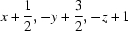
.

**Fig. 1.**
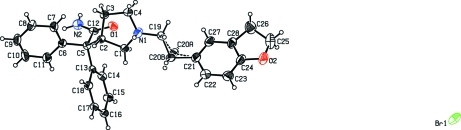
The structure and atom-numbering scheme for (I); displacement ellipsoids are drawn at the 30% probability level.

**Fig. 2.**
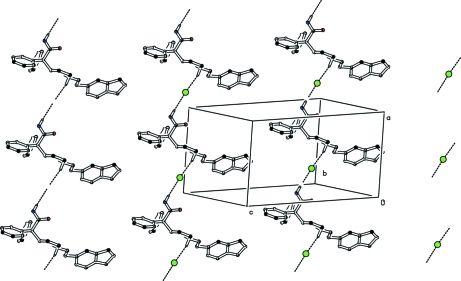
Molecular packing of (I) viewed down the b axis; H-bonds are shown as dashed lines. For the sake of clarity, H atoms, not involved in hydrogen bonds, have been omitted.

## supplementary crystallographic information

### Comment

Darifenacin is a selective muscarinic M3-receptor antagonist that has been evaluated in clinical trials in patients with overative bladder syndrome using a controlled-release formulation (Chapple, 2004; Croom & Keating, 2004; Haab *et al.*, 2004; Levin *et al.*, 2008). As no crystal structure of the title compound has yet been published, we have undertaken the single-crystal X-ray diffraction study and report here its results.

The X-ray study confirmed the molecular structure and atomic connectivity for (I), as illustrated in Fig. 1. The bond length of C12—O1 [1.222 (5) Å] confirms the double bond character for amide group, as evidenced by Allen *et al.* (1987). The C—N and C—-O bond lengths in the pyrrolidine and furan rings are comparable to the related literature values (Selvanayagam *et al.*, 2005).

The pyrrolidine ring adopts an envelope conformation with puckering parameters q_2_ = 0.213 (4) Å and φ = 34.9 (4)° (Cremer & Pople, 1975). Atom C1 deviates by 0.332 (4) Å from the least-squares plane through the remaining four atoms C1—C4 of the ring. The five membered furan ring in the benzofuran system also adopts an envelope conformation with puckering parameters q_2_ = 0.162 (7) Å and φ = 85.9 (8)°. Atom C26 deviate -0.232 (7) Å from the least-squares plane through the remaining four atoms (C24/O2/C25/C27) of the ring.

The dihedral angle between the fused benzene and furan rings of the benzofuran system is 4.6 (2)°. The best plane of the pyrrolidine ring and benzofuran system make a dihedral angle of 44.5 (2)°. Two phenyl rings (A and B) are oriented with a dihedral angle of 72.5 (2)°. These two phenyl rings make a dihedral angle of 33.1 (2) and 82.8 (2)°, respectively with pyrroldine ring.

An intramolecular C—H···O interaction is observed in (I) (Table 2). In the crystal packing, the H1 and H2NB atoms bonded to N1 and N2 forms a strong N—H···Br hydrogen bonds which leads a C(10) and C(9) chain motif in the unit cell running in the opposite directions (Fig. 2). In addition to this the molecular packing is further stabilized by strong C—H···Br hydrogen bonds and C—H···O interactions (Table 2).

### Experimental

In order to obtain crystals suitable for X-ray study, darifenacin hydrobromide was dissolved in a methanol-water solution (80:20v/v); the solvents were then allowed to evaporate slowly.

### Refinement

Atoms H2NA and H2NB were located from a difference Fourier map; the remaining H atoms were positioned geometrically and were treated as riding on their parent C and N atoms  with C—H distances of 0.93–0.97 Å, N—H distance of 0.91 Å and with *U*_iso_= 1.2*U*_eq_ (C or N) for other H. Atom C20 was found to be disordered over two positions. The occupancies were kept fixed at 0.34 and 0.66 during the last cycles of refinement.

### Crystal data

**Table:**

C_28_H_31_N_2_O_2_^+^·Br^−^	*F*(000) = 1056
*M**_r_* = 507.46	*D*_x_ = 1.379 Mg m^−^^3^
Orthorhombic, *P*2_1_2_1_2_1_	Mo *K*α radiation, λ = 0.71073 Å
Hall symbol: P 2ac 2ab	Cell parameters from 18484 reflections
*a* = 10.2632 (7) Å	θ = 2.1–24.9°
*b* = 10.9525 (8) Å	µ = 1.71 mm^−^^1^
*c* = 21.7459 (16) Å	*T* = 293 K
*V* = 2444.4 (3) Å^3^	Block, colourless
*Z* = 4	0.24 × 0.22 × 0.20 mm

### Data collection

**Table:**

Bruker SMART APEX CCD area-detector diffractometer	4703 reflections with *I* > 2σ(*I*)
Radiation source: fine-focus sealed tube	*R*_int_ = 0.065
graphite	θ_max_ = 28.0°, θ_min_ = 1.9°
ω scans	*h* = −13→13
27916 measured reflections	*k* = −14→14
5777 independent reflections	*l* = −28→28

### Refinement

**Table:**

Refinement on *F*^2^	Secondary atom site location: difference Fourier map
Least-squares matrix: full	Hydrogen site location: inferred from neighbouring sites
*R*[*F*^2^ > 2σ(*F*^2^)] = 0.054	H atoms treated by a mixture of independent and constrained refinement
*wR*(*F*^2^) = 0.121	*w* = 1/[σ^2^(*F*_o_^2^) + (0.030*P*)^2^ + 2.3972*P*] where *P* = (*F*_o_^2^ + 2*F*_c_^2^)/3
*S* = 1.07	(Δ/σ)_max_ < 0.001
5777 reflections	Δρ_max_ = 0.55 e Å^−^^3^
315 parameters	Δρ_min_ = −0.84 e Å^−^^3^
2 restraints	Absolute structure: Flack (1983), 2471 Friedel pairs
Primary atom site location: structure-invariant direct methods	Flack parameter: 0.005 (13)

### Special details

**Table:**

Geometry. All e.s.d.'s (except the e.s.d. in the dihedral angle between two l.s. planes) are estimated using the full covariance matrix. The cell e.s.d.'s are taken into account individually in the estimation of e.s.d.'s in distances, angles and torsion angles; correlations between e.s.d.'s in cell parameters are only used when they are defined by crystal symmetry. An approximate (isotropic) treatment of cell e.s.d.'s is used for estimating e.s.d.'s involving l.s. planes.
Refinement. Refinement of *F*^2^ against ALL reflections. The weighted *R*-factor *wR* and goodness of fit *S* are based on *F*^2^, conventional *R*-factors *R* are based on *F*, with *F* set to zero for negative *F*^2^. The threshold expression of *F*^2^ > σ(*F*^2^) is used only for calculating *R*-factors(gt) *etc*. and is not relevant to the choice of reflections for refinement. *R*-factors based on *F*^2^ are statistically about twice as large as those based on *F*, and *R*- factors based on ALL data will be even larger.

### Fractional atomic coordinates and isotropic or equivalent isotropic displacement parameters (Å^2^)

**Table:**

	*x*	*y*	*z*	*U*_iso_*/*U*_eq_	Occ. (<1)
Br1	0.20755 (4)	0.88262 (6)	0.09168 (2)	0.07287 (18)	
O1	0.8147 (3)	0.9481 (3)	1.04271 (14)	0.0553 (8)	
O2	0.2700 (4)	0.7835 (4)	0.66039 (17)	0.0893 (13)	
N1	0.4854 (5)	0.8538 (3)	1.00332 (19)	0.0796 (14)	
H1	0.4034	0.8587	1.0190	0.096*	
N2	0.9041 (4)	1.0090 (4)	1.1310 (2)	0.0599 (10)	
H2NA	0.899 (6)	1.047 (5)	1.1657 (14)	0.08 (2)*	
H2NB	0.979 (3)	0.977 (5)	1.124 (3)	0.088 (19)*	
C1	0.5303 (4)	0.9721 (3)	1.02376 (18)	0.0430 (9)	
H1A	0.4634	1.0334	1.0175	0.052*	
H1B	0.6079	0.9963	1.0014	0.052*	
C2	0.5605 (3)	0.9574 (3)	1.0923 (2)	0.0389 (8)	
H2	0.4809	0.9792	1.1146	0.047*	
C3	0.5793 (4)	0.8189 (4)	1.1003 (2)	0.0475 (9)	
H3A	0.6706	0.7999	1.1068	0.057*	
H3B	0.5299	0.7897	1.1354	0.057*	
C4	0.5312 (6)	0.7607 (4)	1.0422 (2)	0.0686 (14)	
H4A	0.6014	0.7163	1.0224	0.082*	
H4B	0.4615	0.7037	1.0514	0.082*	
C5	0.6692 (3)	1.0458 (3)	1.11486 (17)	0.0362 (8)	
C6	0.6657 (3)	1.0607 (4)	1.18514 (18)	0.0426 (9)	
C7	0.6340 (5)	0.9655 (5)	1.2246 (2)	0.0617 (12)	
H7	0.6143	0.8890	1.2085	0.074*	
C8	0.6316 (6)	0.9834 (6)	1.2875 (2)	0.0774 (16)	
H8	0.6084	0.9190	1.3131	0.093*	
C9	0.6623 (6)	1.0930 (6)	1.3127 (2)	0.0794 (17)	
H9	0.6607	1.1041	1.3551	0.095*	
C10	0.6950 (7)	1.1847 (6)	1.2750 (2)	0.0826 (17)	
H10	0.7169	1.2597	1.2921	0.099*	
C11	0.6975 (5)	1.1726 (4)	1.2122 (2)	0.0611 (11)	
H11	0.7202	1.2388	1.1877	0.073*	
C12	0.8038 (4)	0.9953 (3)	1.0934 (2)	0.0411 (8)	
C13	0.6455 (3)	1.1693 (3)	1.08343 (16)	0.0351 (7)	
C14	0.7313 (4)	1.2233 (4)	1.04213 (18)	0.0448 (9)	
H14	0.8116	1.1868	1.0346	0.054*	
C15	0.6995 (5)	1.3297 (4)	1.0122 (2)	0.0540 (10)	
H15	0.7576	1.3626	0.9838	0.065*	
C16	0.5835 (4)	1.3883 (4)	1.0233 (2)	0.0539 (10)	
H16	0.5631	1.4611	1.0034	0.065*	
C17	0.4976 (4)	1.3360 (4)	1.06486 (19)	0.0516 (10)	
H17	0.4181	1.3738	1.0727	0.062*	
C18	0.5280 (4)	1.2300 (4)	1.0944 (2)	0.0476 (9)	
H18	0.4693	1.1974	1.1225	0.057*	
C19	0.4492 (5)	0.8315 (4)	0.94064 (19)	0.0540 (10)	
H19A	0.3583	0.8072	0.9412	0.065*	
H19B	0.4983	0.7606	0.9274	0.065*	
C20A	0.4620 (17)	0.9225 (13)	0.8917 (6)	0.061 (4)	0.34
H20A	0.5507	0.9234	0.8762	0.073*	0.34
H20B	0.4417	1.0031	0.9075	0.073*	0.34
C20B	0.3745 (7)	0.9232 (6)	0.9058 (3)	0.0522 (15)	0.66
H20C	0.4175	1.0019	0.9090	0.063*	0.66
H20D	0.2882	0.9310	0.9235	0.063*	0.66
C21	0.3624 (6)	0.8874 (5)	0.8370 (2)	0.0757 (15)	
C22	0.2571 (6)	0.9394 (5)	0.8092 (3)	0.0838 (19)	
H22	0.2082	0.9963	0.8310	0.101*	
C23	0.2203 (7)	0.9105 (5)	0.7496 (3)	0.0851 (17)	
H23	0.1490	0.9477	0.7310	0.102*	
C24	0.2931 (6)	0.8250 (5)	0.7192 (2)	0.0676 (13)	
C25	0.3778 (7)	0.7098 (8)	0.6432 (3)	0.101 (2)	
H25A	0.4329	0.7537	0.6145	0.121*	
H25B	0.3474	0.6358	0.6233	0.121*	
C26	0.4540 (6)	0.6781 (7)	0.7004 (3)	0.0924 (19)	
H26A	0.5470	0.6875	0.6941	0.111*	
H26B	0.4357	0.5956	0.7142	0.111*	
C27	0.4011 (5)	0.7728 (5)	0.7451 (2)	0.0620 (12)	
C28	0.4357 (6)	0.8019 (5)	0.8044 (2)	0.0731 (15)	
H28	0.5075	0.7649	0.8227	0.088*	

### Atomic displacement parameters (Å^2^)

**Table:**

	*U*^11^	*U*^22^	*U*^33^	*U*^12^	*U*^13^	*U*^23^
Br1	0.04001 (18)	0.1050 (4)	0.0736 (3)	−0.0003 (3)	−0.0002 (2)	0.0401 (3)
O1	0.0539 (18)	0.0521 (17)	0.0601 (18)	0.0106 (14)	0.0124 (14)	−0.0081 (14)
O2	0.105 (3)	0.098 (3)	0.065 (2)	−0.006 (3)	−0.041 (2)	−0.005 (2)
N1	0.133 (4)	0.044 (2)	0.062 (2)	−0.029 (2)	−0.040 (3)	0.0073 (18)
N2	0.0346 (19)	0.075 (3)	0.070 (3)	0.0114 (19)	0.0007 (18)	−0.004 (2)
C1	0.049 (2)	0.0344 (19)	0.045 (2)	−0.0030 (16)	−0.0053 (17)	−0.0020 (16)
C2	0.0372 (17)	0.0395 (19)	0.0400 (19)	−0.0023 (15)	0.0002 (17)	0.0062 (17)
C3	0.055 (2)	0.042 (2)	0.046 (2)	−0.0027 (18)	0.0002 (19)	0.0074 (18)
C4	0.108 (4)	0.039 (2)	0.058 (3)	0.012 (3)	−0.013 (3)	0.001 (2)
C5	0.0321 (17)	0.0388 (19)	0.0378 (19)	−0.0017 (14)	0.0028 (13)	−0.0005 (15)
C6	0.0356 (18)	0.051 (2)	0.041 (2)	0.0044 (16)	−0.0004 (15)	0.0007 (17)
C7	0.087 (3)	0.060 (3)	0.039 (2)	−0.002 (3)	0.003 (2)	0.001 (2)
C8	0.113 (4)	0.081 (4)	0.039 (3)	0.012 (3)	−0.002 (3)	0.009 (3)
C9	0.095 (4)	0.106 (5)	0.037 (2)	0.033 (3)	−0.006 (2)	−0.015 (3)
C10	0.098 (4)	0.094 (4)	0.056 (3)	0.018 (4)	−0.023 (3)	−0.036 (3)
C11	0.071 (3)	0.060 (3)	0.053 (3)	−0.006 (3)	−0.007 (2)	−0.005 (2)
C12	0.0372 (17)	0.0320 (17)	0.054 (2)	0.0011 (15)	0.008 (2)	0.0026 (17)
C13	0.0376 (16)	0.0329 (17)	0.0347 (18)	−0.0025 (13)	−0.0019 (14)	−0.0049 (15)
C14	0.039 (2)	0.041 (2)	0.054 (2)	−0.0039 (16)	0.0063 (17)	−0.0055 (17)
C15	0.061 (2)	0.042 (2)	0.059 (2)	−0.013 (2)	0.003 (2)	0.0085 (18)
C16	0.071 (3)	0.038 (2)	0.052 (2)	0.002 (2)	−0.014 (2)	−0.003 (2)
C17	0.059 (2)	0.046 (2)	0.050 (2)	0.0158 (19)	−0.0084 (19)	−0.0079 (18)
C18	0.0421 (19)	0.055 (2)	0.046 (2)	0.0082 (17)	0.0016 (18)	0.000 (2)
C19	0.070 (3)	0.041 (2)	0.051 (2)	0.002 (2)	−0.012 (2)	−0.0066 (19)
C20A	0.071 (9)	0.058 (8)	0.053 (8)	−0.014 (7)	−0.012 (7)	0.015 (6)
C20B	0.058 (4)	0.041 (3)	0.058 (4)	0.005 (3)	−0.009 (4)	0.003 (3)
C21	0.123 (4)	0.049 (3)	0.055 (3)	−0.007 (3)	−0.026 (3)	0.006 (2)
C22	0.114 (5)	0.067 (3)	0.071 (4)	0.021 (3)	−0.013 (3)	0.000 (3)
C23	0.094 (4)	0.071 (4)	0.090 (4)	0.016 (3)	−0.038 (4)	−0.009 (3)
C24	0.077 (3)	0.068 (3)	0.058 (3)	−0.010 (3)	−0.026 (3)	0.012 (2)
C25	0.098 (5)	0.141 (7)	0.063 (4)	−0.020 (5)	−0.001 (3)	−0.021 (4)
C26	0.079 (4)	0.105 (5)	0.093 (4)	0.002 (3)	−0.018 (3)	−0.022 (4)
C27	0.061 (3)	0.070 (3)	0.055 (3)	−0.013 (2)	−0.017 (2)	0.002 (2)
C28	0.084 (4)	0.066 (3)	0.070 (3)	0.004 (3)	−0.037 (3)	0.005 (3)

### Geometric parameters (Å, °)

**Table:**

O1—C12	1.222 (5)	C13—C18	1.397 (5)
O2—C24	1.378 (6)	C14—C15	1.374 (6)
O2—C25	1.420 (8)	C14—H14	0.9300
N1—C4	1.406 (6)	C15—C16	1.375 (7)
N1—C19	1.434 (5)	C15—H15	0.9300
N1—C1	1.446 (5)	C16—C17	1.387 (6)
N1—H1	0.9100	C16—H16	0.9300
N2—C12	1.324 (6)	C17—C18	1.363 (6)
N2—H2NA	0.863 (10)	C17—H17	0.9300
N2—H2NB	0.860 (10)	C18—H18	0.9300
C1—C2	1.530 (6)	C19—C20A	1.464 (13)
C1—H1A	0.9700	C19—C20B	1.473 (7)
C1—H1B	0.9700	C19—H19A	0.9700
C2—C3	1.539 (5)	C19—H19B	0.9700
C2—C5	1.557 (5)	C20A—C21	1.614 (14)
C2—H2	0.9800	C20A—H20A	0.9700
C3—C4	1.499 (6)	C20A—H20B	0.9700
C3—H3A	0.9700	C20B—C21	1.551 (9)
C3—H3B	0.9700	C20B—H20C	0.9700
C4—H4A	0.9700	C20B—H20D	0.9700
C4—H4B	0.9700	C21—C22	1.363 (8)
C5—C13	1.535 (5)	C21—C28	1.395 (8)
C5—C6	1.537 (5)	C22—C23	1.388 (8)
C5—C12	1.561 (5)	C22—H22	0.9300
C6—C7	1.389 (6)	C23—C24	1.368 (8)
C6—C11	1.398 (6)	C23—H23	0.9300
C7—C8	1.381 (7)	C24—C27	1.368 (7)
C7—H7	0.9300	C25—C26	1.511 (8)
C8—C9	1.357 (8)	C25—H25A	0.9700
C8—H8	0.9300	C25—H25B	0.9700
C9—C10	1.338 (8)	C26—C27	1.522 (8)
C9—H9	0.9300	C26—H26A	0.9700
C10—C11	1.372 (6)	C26—H26B	0.9700
C10—H10	0.9300	C27—C28	1.375 (7)
C11—H11	0.9300	C28—H28	0.9300
C13—C14	1.390 (5)		
			
C24—O2—C25	107.4 (4)	C14—C15—C16	121.3 (4)
C4—N1—C19	122.4 (4)	C14—C15—H15	119.4
C4—N1—C1	111.0 (4)	C16—C15—H15	119.4
C19—N1—C1	121.8 (4)	C15—C16—C17	118.2 (4)
C4—N1—H1	97.3	C15—C16—H16	120.9
C19—N1—H1	97.3	C17—C16—H16	120.9
C1—N1—H1	97.3	C18—C17—C16	120.9 (4)
C12—N2—H2NA	123 (4)	C18—C17—H17	119.6
C12—N2—H2NB	122 (4)	C16—C17—H17	119.6
H2NA—N2—H2NB	114 (6)	C17—C18—C13	121.5 (4)
N1—C1—C2	105.6 (3)	C17—C18—H18	119.2
N1—C1—H1A	110.6	C13—C18—H18	119.2
C2—C1—H1A	110.6	N1—C19—C20A	123.5 (6)
N1—C1—H1B	110.6	N1—C19—C20B	120.5 (4)
C2—C1—H1B	110.6	C20A—C19—C20B	37.7 (7)
H1A—C1—H1B	108.7	N1—C19—H19A	106.4
C1—C2—C3	103.9 (3)	C20A—C19—H19A	106.4
C1—C2—C5	112.8 (3)	C20B—C19—H19A	72.2
C3—C2—C5	119.1 (3)	N1—C19—H19B	106.4
C1—C2—H2	106.8	C20A—C19—H19B	106.4
C3—C2—H2	106.8	C20B—C19—H19B	131.6
C5—C2—H2	106.8	H19A—C19—H19B	106.5
C4—C3—C2	106.4 (4)	C19—C20A—C21	108.4 (9)
C4—C3—H3A	110.5	C19—C20A—H20A	110.0
C2—C3—H3A	110.5	C21—C20A—H20A	110.0
C4—C3—H3B	110.5	C19—C20A—H20B	110.0
C2—C3—H3B	110.5	C21—C20A—H20B	110.0
H3A—C3—H3B	108.6	H20A—C20A—H20B	108.4
N1—C4—C3	108.0 (4)	C19—C20B—C21	111.4 (5)
N1—C4—H4A	110.1	C19—C20B—H20C	109.3
C3—C4—H4A	110.1	C21—C20B—H20C	109.3
N1—C4—H4B	110.1	C19—C20B—H20D	109.3
C3—C4—H4B	110.1	C21—C20B—H20D	109.3
H4A—C4—H4B	108.4	H20C—C20B—H20D	108.0
C13—C5—C6	110.2 (3)	C22—C21—C28	118.9 (5)
C13—C5—C2	107.1 (3)	C22—C21—C20B	112.6 (6)
C6—C5—C2	111.3 (3)	C28—C21—C20B	128.1 (5)
C13—C5—C12	108.6 (3)	C22—C21—C20A	136.8 (8)
C6—C5—C12	110.8 (3)	C28—C21—C20A	101.1 (8)
C2—C5—C12	108.6 (3)	C20B—C21—C20A	34.8 (6)
C7—C6—C11	116.9 (4)	C21—C22—C23	122.3 (6)
C7—C6—C5	122.7 (4)	C21—C22—H22	118.8
C11—C6—C5	120.4 (4)	C23—C22—H22	118.8
C8—C7—C6	120.6 (5)	C24—C23—C22	117.3 (5)
C8—C7—H7	119.7	C24—C23—H23	121.3
C6—C7—H7	119.7	C22—C23—H23	121.3
C9—C8—C7	121.5 (5)	C23—C24—C27	122.0 (5)
C9—C8—H8	119.3	C23—C24—O2	125.4 (5)
C7—C8—H8	119.3	C27—C24—O2	112.6 (5)
C10—C9—C8	118.3 (5)	O2—C25—C26	108.4 (5)
C10—C9—H9	120.8	O2—C25—H25A	110.0
C8—C9—H9	120.8	C26—C25—H25A	110.0
C9—C10—C11	122.8 (5)	O2—C25—H25B	110.0
C9—C10—H10	118.6	C26—C25—H25B	110.0
C11—C10—H10	118.6	H25A—C25—H25B	108.4
C10—C11—C6	120.0 (5)	C25—C26—C27	100.7 (5)
C10—C11—H11	120.0	C25—C26—H26A	111.6
C6—C11—H11	120.0	C27—C26—H26A	111.6
O1—C12—N2	122.4 (4)	C25—C26—H26B	111.6
O1—C12—C5	120.1 (4)	C27—C26—H26B	111.6
N2—C12—C5	117.6 (3)	H26A—C26—H26B	109.4
C14—C13—C18	117.1 (3)	C24—C27—C28	119.9 (5)
C14—C13—C5	124.2 (3)	C24—C27—C26	108.1 (4)
C18—C13—C5	118.7 (3)	C28—C27—C26	131.7 (5)
C15—C14—C13	121.1 (4)	C27—C28—C21	119.5 (5)
C15—C14—H14	119.5	C27—C28—H28	120.2
C13—C14—H14	119.5	C21—C28—H28	120.2
			
C4—N1—C1—C2	−23.2 (6)	C5—C13—C14—C15	175.0 (4)
C19—N1—C1—C2	−179.2 (5)	C13—C14—C15—C16	1.9 (6)
N1—C1—C2—C3	20.9 (4)	C14—C15—C16—C17	−1.2 (6)
N1—C1—C2—C5	151.3 (4)	C15—C16—C17—C18	0.7 (6)
C1—C2—C3—C4	−12.2 (5)	C16—C17—C18—C13	−0.8 (6)
C5—C2—C3—C4	−138.7 (4)	C14—C13—C18—C17	1.4 (6)
C19—N1—C4—C3	171.2 (5)	C5—C13—C18—C17	−175.7 (4)
C1—N1—C4—C3	15.3 (7)	C4—N1—C19—C20A	−149.3 (10)
C2—C3—C4—N1	−1.1 (6)	C1—N1—C19—C20A	4.0 (12)
C1—C2—C5—C13	40.3 (4)	C4—N1—C19—C20B	166.0 (6)
C3—C2—C5—C13	162.5 (4)	C1—N1—C19—C20B	−40.7 (8)
C1—C2—C5—C6	160.8 (3)	N1—C19—C20A—C21	−157.4 (7)
C3—C2—C5—C6	−77.0 (5)	C20B—C19—C20A—C21	−59.4 (9)
C1—C2—C5—C12	−76.9 (4)	N1—C19—C20B—C21	172.5 (5)
C3—C2—C5—C12	45.3 (5)	C20A—C19—C20B—C21	65.9 (11)
C13—C5—C6—C7	154.5 (4)	C19—C20B—C21—C22	156.0 (6)
C2—C5—C6—C7	35.8 (5)	C19—C20B—C21—C28	−16.8 (10)
C12—C5—C6—C7	−85.2 (5)	C19—C20B—C21—C20A	−62.4 (10)
C13—C5—C6—C11	−26.7 (5)	C19—C20A—C21—C22	118.0 (10)
C2—C5—C6—C11	−145.4 (4)	C19—C20A—C21—C28	−83.9 (11)
C12—C5—C6—C11	93.6 (4)	C19—C20A—C21—C20B	61.1 (9)
C11—C6—C7—C8	1.5 (7)	C28—C21—C22—C23	0.0 (9)
C5—C6—C7—C8	−179.7 (5)	C20B—C21—C22—C23	−173.6 (6)
C6—C7—C8—C9	−1.3 (9)	C20A—C21—C22—C23	155.2 (10)
C7—C8—C9—C10	0.2 (9)	C21—C22—C23—C24	1.0 (10)
C8—C9—C10—C11	0.6 (10)	C22—C23—C24—C27	−2.5 (9)
C9—C10—C11—C6	−0.4 (9)	C22—C23—C24—O2	178.9 (6)
C7—C6—C11—C10	−0.7 (7)	C25—O2—C24—C23	171.6 (6)
C5—C6—C11—C10	−179.5 (5)	C25—O2—C24—C27	−7.1 (7)
C13—C5—C12—O1	−77.7 (4)	C24—O2—C25—C26	15.4 (7)
C6—C5—C12—O1	161.0 (4)	O2—C25—C26—C27	−16.7 (7)
C2—C5—C12—O1	38.5 (5)	C23—C24—C27—C28	3.0 (8)
C13—C5—C12—N2	100.3 (4)	O2—C24—C27—C28	−178.2 (5)
C6—C5—C12—N2	−20.9 (5)	C23—C24—C27—C26	177.2 (6)
C2—C5—C12—N2	−143.5 (4)	O2—C24—C27—C26	−4.0 (6)
C6—C5—C13—C14	122.6 (4)	C25—C26—C27—C24	12.5 (6)
C2—C5—C13—C14	−116.2 (4)	C25—C26—C27—C28	−174.3 (6)
C12—C5—C13—C14	1.0 (5)	C24—C27—C28—C21	−1.9 (8)
C6—C5—C13—C18	−60.5 (4)	C26—C27—C28—C21	−174.5 (6)
C2—C5—C13—C18	60.8 (4)	C22—C21—C28—C27	0.5 (9)
C12—C5—C13—C18	177.9 (3)	C20B—C21—C28—C27	172.9 (6)
C18—C13—C14—C15	−2.0 (6)	C20A—C21—C28—C27	−162.5 (7)

### Hydrogen-bond geometry (Å, °)

**Table:**

*D*—H···*A*	*D*—H	H···*A*	*D*···*A*	*D*—H···*A*
N1—H1···Br1^i^	0.91	2.57	3.453 (6)	164
N2—H2NB···Br1^ii^	0.86 (1)	2.66 (1)	3.514 (4)	175 (5)
C18—H18···O2^iii^	0.93	2.60	3.382 (6)	142
C19—H19B···Br1^iv^	0.97	2.69	3.609 (5)	158
C4—H4A···Br1^iv^	0.97	2.92	3.770 (5)	147
C1—H1B···O1	0.97	2.37	2.959 (5)	119

Symmetry codes: (i) *x*, *y*, *z*+1; (ii) *x*+1, *y*, *z*+1; (iii) −*x*+1/2, −*y*+2, *z*+1/2; (iv) *x*+1/2, −*y*+3/2, −*z*+1.
